# Arylamine N-Acetyltransferases in Mycobacteria

**DOI:** 10.2174/138920008784892100

**Published:** 2008-07

**Authors:** Edith Sim, James Sandy, Dimitrios Evangelopoulos, Elizabeth Fullam, Sanjib Bhakta, Isaac Westwood, Anna Krylova, Nathan Lack, Martin Noble

**Affiliations:** 1 Department of Pharmacology, University of Oxford, Mansfield Road, Oxford OX1 3QT, UK; 2 School of Biological and Chemical Sciences, Faculty of Science, Birkbeck University of London, Malet Street, London WC1E 7HX, United Kingdom; 3 Department of Molecular Biophysics, University of Oxford, South Parks Road, Oxford OX1 3QU, UK

**Keywords:** Isoniazid, tuberculosis, *M. smegmatis*, *M. marinum*, arylamine.

## Abstract

Polymorphic Human arylamine N-acetyltransferase (NAT2) inactivates the anti-tubercular drug isoniazid by acetyltransfer from acetylCoA. There are active NAT proteins encoded by homologous genes in mycobacteria including *M. tuberculosis, M. bovis* BCG, *M. smegmatis *and *M. marinum*. Crystallographic structures of NATs from *M. smegmatis *and *M. marinum, *as native enzymes and with isoniazid bound share a similar fold with the first NAT structure, *Salmonella typhimurium *NAT. There are three approximately equal domains and an active site essential catalytic triad of cysteine, histidine and aspartate in the first two domains. An acetyl group from acetylCoA is transferred to cysteine and then to the acetyl acceptor e.g. isoniazid. **M. marinum **NAT binds CoA in a more open mode compared with CoA binding to human NAT2. The structure of mycobacterial NAT may promote its role in synthesis of cell wall lipids, identified through gene deletion studies. NAT protein is essential for survival of *M. bovis *BCG in macrophage as are the proteins encoded by other genes in the same gene cluster (*hsaA-D*). HsaA-D degrade cholesterol, essential for mycobacterial survival inside macrophage. Nat expression remains to be fully understood but is co-ordinated with *hsaA-D *and other stress response genes in mycobacteria.

Amide synthase genes in the streptomyces are also *nat *homologues. The amide synthases are predicted to catalyse intramolecular amide bond formation and creation of cyclic molecules, e.g. geldanamycin. Lack of conservation of the CoA binding cleft residues of *M.* *marinum *NAT suggests the amide synthase reaction mechanism does not involve a soluble CoA intermediate during amide formation and ring closure.

## INTRODUCTION

The relationship between arylamine N-acetyltransferases and the mycobacteria has been established for many years [1]. The first identification of pharmacogenetic variation in the metabolism of isoniazid provided an immediate link with the mycobacteria as isoniazid is still the front line drug for treatment of tuberculosis [2,3].

NATs in bacteria were very important in establishing the active site catalytic triad as the mechanism whereby cysteine could be activated [4] to participate in transacetylation as the first stage in the reaction catalysed by arylamine N-acetyltransferases and covered elsewhere in this volume. The existence of the catalytic triad is essential for acetyltransferase activity in all organisms in which NAT has been found [5-9]. NATs and their homologues have now been characterized from a number of bacteria [10-15]. The NAT from *Salmonella typhimurium* was the first to be characterized both in terms of enzymic activity and three-dimensional structure [4,16-18]. 

## NAT IN MYCOBACTERIA

Amongst the earliest bacterial NATs to be identified and characterized at the structural level was the NAT from *Mycobacterium smegmatis*. The early work on mycobacterial *nat* genes co-incided with the completion of the genome of *Mycobacterium tuberculosis* the virulent laboratory strain H37Rv [19,20]. These studies have now been accompanied by the completion of many other mycobacterial genomes ([http://genolist.pasteur.fr/TubercuList/]). Through these and related studies ([http://www.tigr.org] [http://www.sanger. ac.uk/Projects/Microbes/]) it was identified that *nat* genes were present in a range of mycobacteria (Tables **1** and **2**). From the studies of NATs which have been found in different mycobacteria, the level of similarity has been established and as for the other NAT sequence comparisons, the level of identity is least in the third domain Fig. (**1**). So far mycobacterial *nat* genes have been identified in the slow growing mycobacteria including *M. tuberculosis* and also the non-pathogenic model strain *Mycobacterium bovis* BCG. A homologous gene is also present in the fast growers such as *Mycobacterium smegmatis*. In contrast, *Mycobacterium leprae* is missing a third of its genome including the *nat *gene and is unable to live outside cells.

The *nat* genes from *M. tuberculosis* and *M. bovis* BCG are identical (Tables **1** and **2**) and consequently the open reading frames are also identical (Table **2**). It has been established that the *nat *gene is encoded in highly similar operons in *M. bovis* BCG and *M. tuberculosis* [21]. Interestingly this operon has been established to be essential for survival of *M. tuberculosis* within macrophage [22], as has the *nat* gene itself [23]. The NAT protein appears to play an important role in the synthesis of the mycobacterial cell wall in the slow growing mycobacteria and has been suggested to be a target for anti-mycobacterial therapy [23]. Interestingly, the operon appears to be upregulated following stress [24]. The precise role that the *nat* gene product plays in the stress response has not yet been established although a possibility for a metabolic role in relation to CoA intermediate homeostasis cannot be ruled out. Understanding the gene organization in different mycobacteria may help to unravel this role.

## *nat* GENES AND OPERON ORGANIZATION IN DIFFERENT MYCOBACTERIAL SPECIES

The *nat* gene was predicted to be part of an operon in most mycobacterial species [25]. The putative *nat* operon in *M. tuberculosis *H37Rv and in *M. bovis *BCG has been characterized to consist of six genes including *nat *[21] Fig. (**2**). The other genes encode the proteins known as HsaA, HsaD, HsaC, HsaB and there is also a pseudogene [21]. NAT protein has been found to be essential for survival of *M. bovis* BCG inside macrophage cells [23]. HsaA and HsaD are considered to play a part in cholesterol degradation [26], and cholesterol has now been shown to be an essential fuel for mycobacterial survival in host cells [27]. The genes of the operon are required for intracellular survival of *M. tuberculosis* in macrophages [22]. Investigation of the *nat *operon in different mycobacteria will shed light on the function of the novel endogenous pathway encoded by these genes. The availability of the genome sequence from a number of mycobacteria, and the use of bioinformatic tools, has assisted the prediction and comparative analysis of the conservation of the *nat *operon among mycobacteria (Table **1**). 

The gene organization around *nat* has been found to be almost identical in different strains of *M. tuberculosis *(Table **1**), although no pseudogene appears to be present in the F11 or C strains. *M. tuberculosis *strain Haarlem showed 100% conservation of all genes in the cluster compared with H37Rv.

*M. bovis *and *M. bovis *BCG strains both show very high conservation of all of the operon genes in their genome. The conservation of each gene is above 98% and each strain also contains the pseudogene as in *M. tuberculosis *H37Rv.

Other mycobacterial species with a conserved organization around the *nat* gene are the *Mycobacterium avium *paratuberculosis K-10 and 104 strains. All of the homologous genes in these organisms are highly conserved - above 80% with the exception of the *nat* gene which is only 67% for strain K-10 and 68% for strain 104, compared with H37Rv. The pseudogene is not present in *M. avium *strains. 

In other mycobacterial strains, there appear to be separate gene clusters for the *nat *and *hsa *genes. *Mycobacterium marinum *and *Mycobacterium ulcerans*, the closest relatives phylogenetically to the *M. tuberculosis *complex [28] have a *nat* gene in close proximity to *hsa *genes. Although *nat *is separated from the homologues of the other genes found in the putative operon in *M. tuberculosis* H37Rv, the homologues *hsaA, hsaD, hsaC, hsaB *are adjacent to each other and in the same order as in *M. tuberculosis* H37Rv. This pattern of the four *hsa *genes being in an operon has been found in most mycobacterial species and also in the rhodococci which also have mycolic acids in their cell wall [26]. In addition the genes found between *nat *and the *hsa *gene clusters in **M. marinum **and *M. ulcerans* are highly conserved and are likely to encode for proteins with similar function [29,26 and references therein]. 

In the fast-growing soil-dwelling mycobacteria the pattern of the *nat *gene cluster differs. There are genes adjacent to *nat, *encoding open reading frames with putative different function. The *nat *operon in *M. smegmatis *mc^2^ 155 is a putative three gene operon with *nat *being the first gene, followed by a gene that encodes for a transcriptional regulator belonging to the AraC/XylS protein family, and a third gene annotated as encoding dihydrofolate reductase. The same pattern, but with the addition of two more genes has been found in *Mycobacterium gilvum *PYR-GCK and *Mycobacterium vanbaalenii *PYR-1. The additional genes in these latter two strains encode an acetyl-CoA dehydrogenase-like protein, based on homology, and a hypothetical protein. The three genes found in the *Mycobacterium smegmatis *mc^2^ 155 “*nat* operon” exist also in *Mycobacterium sp. *KMS, MCS and JLS strains. The only difference is that the *nat *gene is separated by two more genes (encoding potential dehydrogenases) between *nat *and *araC*. In addition, the dihydrofolate reductase gene homologue is annotated in this genome as a bifunctional deaminase-reductase like protein (Table **1**, Fig. (**2**)).

There is clearly variation of the *nat *gene clusters amongst mycobacteria and work requires to be done to establish unequivocally the nature of the proteins encoded. We can distinguish three different operon patterns. In slow-growing and most pathogenic mycobacteria, including *M. tuberculosis *H37Rv, the *nat *and the *hsa *genes forming one operon. In the relatively faster growing pathogenic close relatives such as *M. ulcerans *and **M. marinum **the *nat *gene cluster is relatively close to the *hsa *genes, whereas in the fast growing environmental non-pathogenic mycobacteria, *nat *belongs to a different gene cluster distinct from that in slow-growers, Fig. (**2**). 

Although the control of gene expression patterns in mycobacteria is not well understood, it is likely to be at the transcriptional level [30]. There are about 190 transcription regulatory proteins annotated in the genome of *M. tuberculosis* [19]. There are single, multiple and overlapping, as well as alternative internal promoters in the genome that play an important role in the transcription of a single gene or operon [31]. The “*nat *operon” in *M. tuberculosis *is part of the *kstR* regulon (stress regulon) that is involved in the lipid metabolism [24]. The same study showed that the *hsa* gene cluster (Fig. (**2**)) seems to belong to the *kstR* regulon in *M. smegmatis *mc^2^ 155, a fast growing organism where the *nat *gene is part of a distinct gene cluster. However, the transcriptional control recognition motif is present upstream of the *nat *gene as well as upsteam of the *hsa* gene cluster in *M. smegmatis, *suggesting that at least under certain conditions *nat *and *hsa *genes are co-ordinately regulated in *M. smegmatis* even though they are in different operons.

Studying the organization and different components of the operons in which *nat *genes are found will help to understand the roles of *nat* and the associated genes in mycobacteria and in actinomycetales.

## GENETIC MODIFICATION AND IDENTIFICATION OF POLYMORPHISMS

In order to understand the role of the *nat* gene in mycobacteria, experiments were carried out on genetically modified strains [20,32,23]. Investigation of possible polymorphisms in *nat* genes in clinical isolates of *M. tuberculosis* were also investigated [33-35]. Initially, the main interest was that the *nat* gene product might have a role in isoniazid resistance in *M. tuberculosis* since it was known that human NAT2 metabolised isoniazid to the therapeutically inactive form *N*-acetylisoniazid [36,37]. Effectively isoniazid is a pro-drug and it was considered that if isoniazid were acetylated within mycobacterial cells that it would not be activated by the *katG* gene product. The activated isoniazid inhibits the synthesis of mycolic acids [38]. In order to test this hypothesis, initially it was important to identify whether the *nat* gene was expressed in *M. tuberculosis*. It was clearly demonstrated that the *nat* gene was transcribed and also that protein was present in cytosols of growing organisms which could be detected with specific antisera against *M. tuberculosis* NAT [32]. The NAT protein was active in acetylation of isoniazid [33]. These studies confirmed that the *nat* gene product was transcribed and active in *M. tuberculosis *and* M. bovis *BCG. Other studies confirmed that the same is true of the *nat* gene product in *M. smegmatis* [20,25,32]. The NAT enzyme from *M. smegmatis* was demonstrated to be able to metabolise isoniazid and when the *nat* gene from *M. smegmatis* was overexpressed in *M. bovis* BCG the susceptibility of the slow growing mycobacterium to isoniazid was decreased as would be expected [20]. 

The corollary experiment in which the *nat* gene is deleted was carried out in *M. bovis* BCG [23] and also *M. smegmatis* [32]. In these studies the sensitivity to isoniazid was increased by up to three fold. 

Investigations of clinically isolated strains of *M. tuberculosis* identified point mutations in the *nat *gene [33,35]. The mutations which were found resulted in the substitution of an arginine residue in place of a glycine residue at position 207 [33] and this mutation was accompanied by a second mutation in some clinical strains in which a tyrosine at position 177 is replaced by a histidine (compare Fig. (**1**). The effect of the Y177H mutation has not been determined directly using recombinant protein studies but recombinant NAT protein from *M. tuberculosis* showed that the G207R mutation resulted in a NAT enzyme with very poor activity [33]. A corresponding mutation introduced into NAT from *M. smegmatis*, does not have such a deleterious effect on enzymic activity [34]. 

Each of the mutations in *nat *was restricted to the same family of clinical isolates of *M. tuberculosis *[35]. There was no clear correlation between these loss-of-function mutations and isoniazid sensitivity, although it would be expected that mutations resulting in loss of function of NAT would improve sensitivity to isoniazid. It was concluded that NAT appears to modulate the effects of other known genetic factors on isoniazid sensitivity of strains of *M. tuberculosis*, but NAT activity it is not directly correlated with isoniazid resistance. A gain of function mutation would be required to induce isoniazid resistance. 

## NAT APPEARS TO PLAY AN ENDOGENOUS ROLE IN MYCOBACTERIA

The clinical isolates were considered to belong to strain families which grow particularly slowly [35], although growth of slow growing mycobacteria can be temperamental *in vitro*.

When the *nat *gene was deleted in *M. smegmatis* [32] and also in *M. bovis* BCG, there were interesting findings suggesting an endogenous role for the NAT enzyme. This has been reviewed extensively [39] and so will be covered in summary here. The growth of the *nat* deleted strains of both *M. bovis* BCG and *M. smegmatis* was shown to be delayed. In *M. bovis* BCG, it was noted that the ultrastructure and susceptibility to antibiotics was altered. The organisms became more susceptible to antibiotics such as gentamycin to which they were normally resistant [23]. It was also noted that the characteristic mycobacterial cell wall lipid components were not present in the *nat* deleted strain of *M. bovis* BCG but complementation with *nat* restored the wild type phenotype. These studies suggested that *nat* has either a direct or an indirect role in cell wall lipid metabolism. It still remains to be determined whether the *nat* gene product affects lipid metabolism directly or whether it interferes with metabolic energy production and reducing equivalents essential for cell wall lipid synthesis. One important feature of these studies was the demonstration that the *nat* gene is essential for survival of *M. bovis* BCG within macrophage. As stated above, this is also true of the other genes which have been identified in the same operon [22].

Specific NAT inhibitors have been identified through high throughput screening of chemical libraries [40,41] and through investigating natural products [42] using the mycobacterial NAT enzymes as targets. These studies have clearly shown that inhibition of NAT activity in *M. bovis* BCG has very similar effects to deleting the *nat* gene. Inhibitors of the other genes in the operon [21] which are essential for survival of *M. tuberculosis* in macrophage have been demonstrated to affect the cell wall lipid composition in a similar fashion to lack of NAT activity [21]. Recently these genes have been suggested to have a role in cholesterol degradation [26]. Since it now appears that cholesterol is essential as a fuel for mycobacteria inside macrophage [27], it is looking increasingly likely that NAT has a role in intermediary metabolism and energy production in mycobacteria inside macrophage but this still needs to be investigated.

## STRUCTURAL STUDIES

The NAT proteins encoded by *nat* genes from *M. bovis* BCG and *M. tuberculosis* are identical (Tables **1** & ** 2**) and although small amounts have been obtained for analytical purposes, insufficient has been generated to allow a full structural characterization [33, presented data^3^].

The sequences of NATs from a range of mycobacteria and mycolata have been compared (Table **2**) and sequence conservation is very high in all of these proteins in the first two domains but less highly conserved in the third domain (Table **2**). In view of the small amount of *M. tuberculosis* NAT protein available to date, NAT from other mycobacterial sources has been used for structural studies. NAT from *M. smegmatis* and NAT from the organism *M. marinum* [43] which is being used as a model for *M. tuberculosis* have been investigated. *M. marinum*, as the name suggests, infects fish and frogs. The mycobacterial NATs which have been generated in mg quantities as recombinant proteins have been compared in relation to their substrate specificity profiles [43]. The specificity profile of *M. marinum* is very similar to NAT from *M. smegmatis* except that p-aminobenzoic acid and procainamide are poorer substrates for the NAT enzyme from *M. marinum*. 

The structure of the NAT from *M. smegmatis* [44] was the first structure of a mycobacterial NAT enzyme to be obtained and the active site catalytic triad is superimposable on the structure of the enzyme from *S. typhimurium *[4] (Fig. (**3**)). 

The amino acids which interact with isoniazid as substrate at the active site have been identified through structure determination following co-crystallisation with both *M. marinum* NAT and with NAT from *M. smegmatis* (Fig. (**4**)). The main residues interacting with isoniazid are entirely conserved in these two proteins (Fig. (**1**)). The structure of NAT from *M. smegmatis* does not differ significantly between the structure with isoniazid bound and the structure of the enzyme alone [7]. The same is true of the NAT enzyme from *M. marinum* [43]. However, when the structure of the NAT enzyme from *M. marinum* was solved, it was clear that there were two binding sites for isoniazid, one was the same as the active site identified in *M. smegmatis* NAT, but the other was at a more exposed surface site. This second site was subsequently shown to correspond to the binding site of the adenine rings in Coenzyme A through a co-crystallisation study of NAT from *M. marinum* with CoA [43].

At around the time that these studies were being done, the structure of a human NAT enzyme with CoA bound was also obtained [9]. The binding sites for CoA in these two enzymes are clearly distinct Fig. (**5**). There have been discussions of the loop region which is present in eukaryotic NAT enzymes between the second and third domains but which is missing in the prokaryotic enzymes [25,45,46]. It has been demonstrated that the loop and also the C-terminus of the human enzyme is folded across the active site cleft [9]. The C-terminus has been demonstrated to be important in determining the activity of the NAT from *S. typhimurium* [17] and also in contributing to specificity of different mammalian isoenzymes [45,47] along with the region around residue 124-129 in the mammalian NAT structures [48,9]. The differences in binding of CoA to human NAT2 and to *M. marinum* NAT (Fig. (**5**)) are likely to result from the interactions with the loop and the C-terminus of the human enzyme which partly occludes the active site cleft (Fig. (**5**)) [43,49].

It has been discussed that the difference in the CoA binding to mycobacterial and human NAT is an evolutionary snap-shot [43]. What is unclear is whether the difference in binding represents the different roles that these enzymes play in their respective organisms.

## BACTERIAL NAT HOMOLOGUES CATALYZING AMIDE SYNTHESIS

Many Streptomyces and related species contain genes homologous to the *nat* genes. These genes encode amide synthase^4^ enzymes which catalyse a ring-closure reaction to yield a large macro-cyclic compound (Fig. (**6**)). Compounds produced are antibacterial, for example Rifamycin from *Amycolatopsis mediterranei *[50], or anti-tumour activity, such as Ansamitocin from *Actinosynnema pretiosum* [51,52].

The genes encoding the amide synthase enzymes are commonly found at the end of a gene cluster encoding Polyketide Synthase enzymes [53-57]. These enzymes build up a large chain in a stepwise manner [58,59]. The amide synthetase enzyme joins the amine end of the chain to the carbonyl which has been attached to an acyl carrier protein enzyme, forming the amide bond, as shown in Fig. (**6**). 

Whilst there has been much analysis of the polyketide synthase enzymes within these gene clusters responsible for the growing acyl chain [54,59,55,60] the enzymes responsible for ring closure which are homologous to the NAT enzymes have been studied to a lesser extent. RifF, from the Rifamycin producing bacterium *A.* *mediterranei*, has been investigated and recombinant enzyme has been produced [14]. Based on homology modelling, it was proposed that the amide synthase RifF would share the same fold as that of the NAT enzymes due to the high sequence similarity [14]. To date, no crystallographic structure of this family of enzymes homologous to the NAT enzymes exists. 

Overall, the amide synthase enzymes show similarity with the NAT enzymes (Fig. (**7**)). The catalytic triad of residues Cys – His – Asp is completely conserved, one of the more obvious differences is that in the NAT enzymes, the PFENL motif (very highly conserved in NATs) is not conserved in the amide synthase enzymes. The amide synthase enzymes have a common sequence of PYD** in place of the PFENL motif. Several residues which have been identified as essential for NAT activity (Arg 9 and Arg 64) [61] are again not completely conserved in the amide synthase enzymes. Leu 24 has been proposed to be essential for stability of the protein due to its interaction with Leu 79 and Val 112 [62], these residues being conserved across the NAT and amide synthetase enzymes. Arg 155 appears to play a role in stabilising the beta barrel domain. Pro 133 is also conserved between the amide synthase and NAT enzymes, possibly contributing towards the conformation of the loop in which it is situated. Additionally, Tyr 190 is completely conserved amongst all enzymes and is situated proximal to the catalytic Asp 122. This residue may play a role in holding the Asp 122 residue in position, maintaining the conformation of the catalytic triad. Numbering is corresponding to [62].

Whilst there are many similarities in the sequences between the NAT enzymes and the amide synthetase enzymes, there are likely to be subtle differences in the structures of the two enzymes just as observed between NATs from different species [12,43,9]. Recent publications have described the mode of binding of CoenzymeA to the prokaryotic *M. marinum* NAT [43] and human NAT2 [9]. The residues involved in CoA binding are not well conserved in the amide synthases. It is likely that given there are two distinct modes of CoA binding to the NAT enzymes, that there will be differences in ligand binding in the amide synthase enzymes. Comparison of the residues involved in CoA binding in the NAT enzymes, with the amide synthase enzymes shows that the necessary residues are not conserved across in the amide synthases. It is likely therefore that there could be an entirely novel mode of binding of ligands within the amide synthetase active site. Given that there are likely to be interactions between the polyketide synthase enzymes and the amide synthases encoded by the gene clusters, protein:protein interactions may play a part in understanding how these enzymes carry out their ring closure reaction. This is a good example of divergent evolution where a similar protein fold can carry out multiple functions.

The NAT field is ripe to begin to answer the questions on the mycobacterial NATs and the amide synthetases with the availability of physical and genetic techniques, identifying the importance of a multi-disciplinary approach embracing systems biology as well as molecular analytical techniques to understand biology.

## Figures and Tables

**Fig. (1). Comparison of the amino acid sequences of NATs from mycobacteria.**
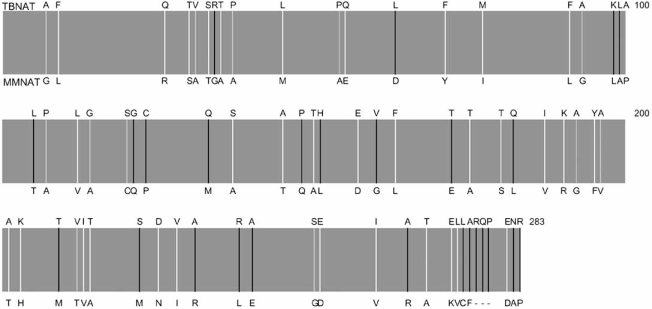
A schematic diagram to show the amino acids which differ between MMNAT and TBNAT based on a ClustalW alignment (http://www.ebi.ac.uk/clustalw) and Espript [63]. The amino acids indicated above the bar are TBNAT residues and the amino acids indicated below the bar are MMNAT residues. White lines indicate residues which are a conserved substitution, grey lines indicate residues which are semi-conserved, black lines indicate residues which are nonconservative and the dashes indicate deletions since MMNAT is shorter than TBNAT. The numbering is based on the TBNAT sequence. Amino acids are indicated by single letters.

**Fig. (2). Comparison of the operon organization of mycobacterial species.**
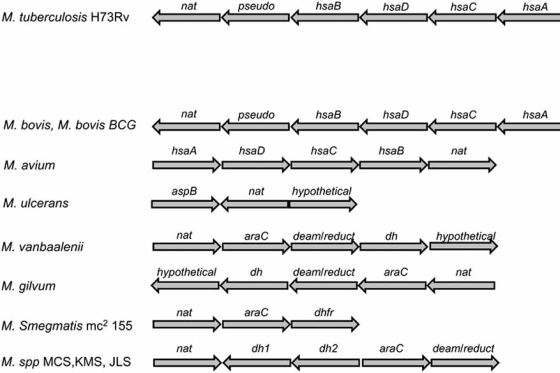
Operon analysis was carried out using the method described in [21] and also [31]. Genes have been named as they are annotated in the relevant genomes: *nat* = arylamine N-acetyltransferase; pseudo = pseudogene; *hsa* gene cluster is named as described in [21] and [26]; *aspB* is an aspartate aminotransferase; *araC* is homologous to the *Escherichia coli* transcription regulator [64]; *deam/reduct* refers to deaminase/reductase gene product; *dh, dh1 and dh2* correspond to homologues of dehydrogenase gene products; *dhfr* = dihydrofolate reductase. The *nat* genes in *M. tuberculosis, M. bovis* and *M. smegmatis,* and also *hsaD* and *hsaC* from *M. tuberculosis* and *M. bovis* encode proteins where enzyme activity has been confirmed. All of the open reading frames encode for putative proteins. See  http://genolist.pasteur.fr/TubercuList/, http://www.tigr.org, http://www.sanger.ac.uk/Projects/Microbes/

**Fig. (3). Overlay of the crystal structures of NATs from bacteria.**
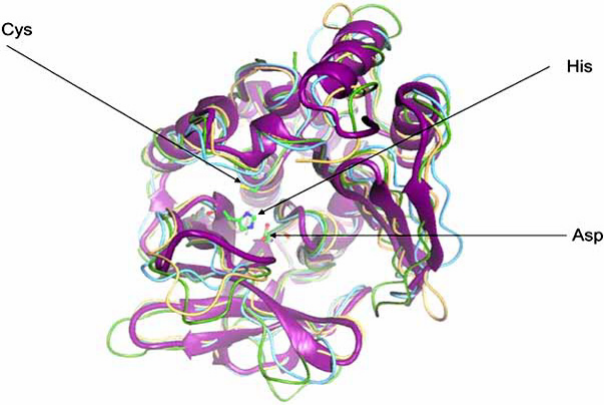
NATs from *M. smegmatis* (pdb code 1gx3), *S. typhimurium* (pdb code 1e2t), P. *aeruginosa* (pdb code 1w4f) and *M. loti* NAT1 (pdb code 2bsz) are shown superimposed, each in a different grey tone. The active site triad residues are shown in ball and stick representation and are indicated by arrows. After [13].

**Fig. (4). Interactions of isoniazid with the active site of NAT from M. smegmatis.**
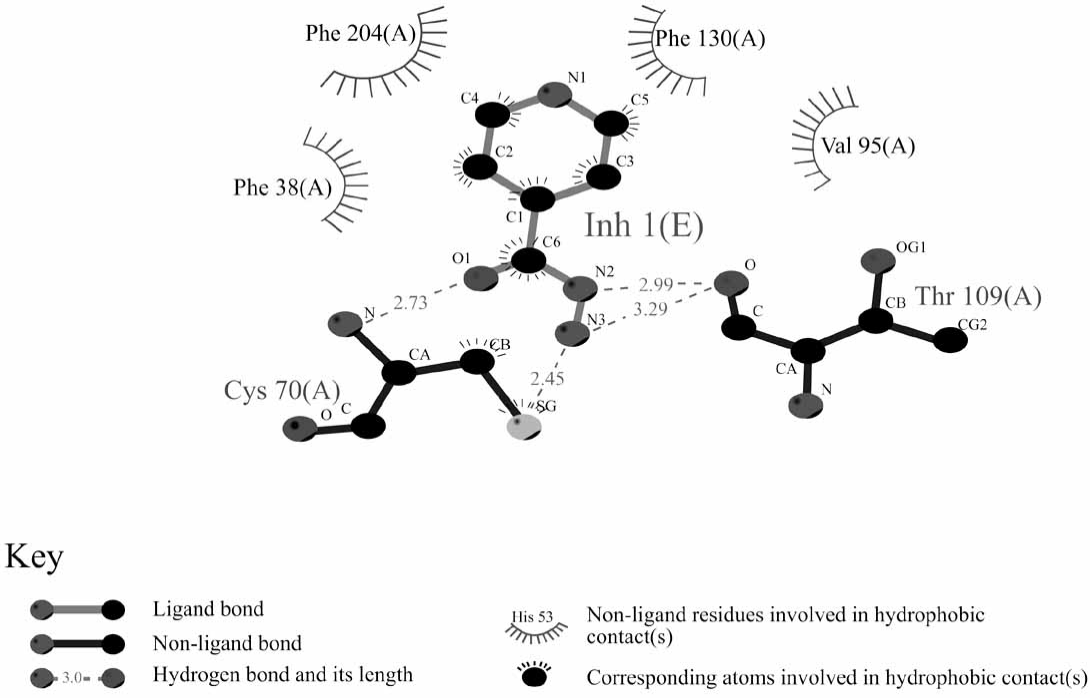
The interactions are shown using Ligplot analysis [65]. Inh1 (E) indicates isoniazid. The residues are indicated by their numbers in *M. smegmatis* NAT. After [7]. An identical Ligplot for *M. marinum* NAT is found in [43].

**Fig. (5). Comparison of the interaction of CoA with NAT from M. marinum and human NAT2.**
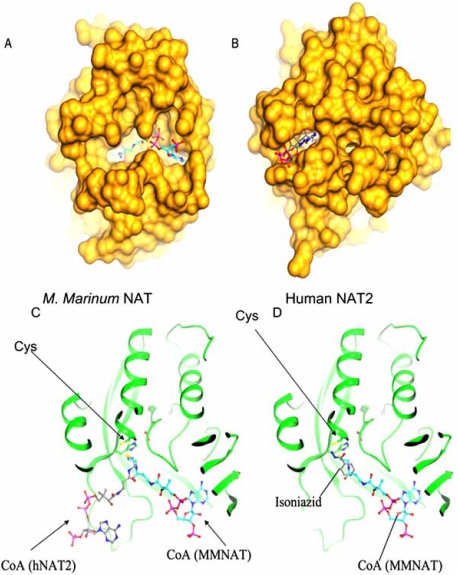
Molecular surface representations of * M. marinum* NAT with CoA bound (pdb 2vfc) (A) with human NAT2 with CoA bound (pdb 2pfr) (B). The CoA is shown in ball and stick representation. Ribbon representation of the binding of *M marinum *NAT (MMNAT) with CoA bound (dark ribbon) compared with CoA bound to human NAT2(hNAT2) (light ribbon). The structures have been overlaid and the CoA molecules are shown in ball and stick representation, as are the residues of the active site triad (Cys indicated by an arrow) (C). Frame (D) shows the location of isoniazid in the active site, in relation to the position of CoA in the structure of *M. marinum *NAT. Isoniazid and CoA are shown in ball and stick representation and the active site resides are just visible, with Cys being indicated by an arrow. After [43].

**Fig. (6). The reactions leading to the synthesis of Geldanamycin by amide ring closure.**
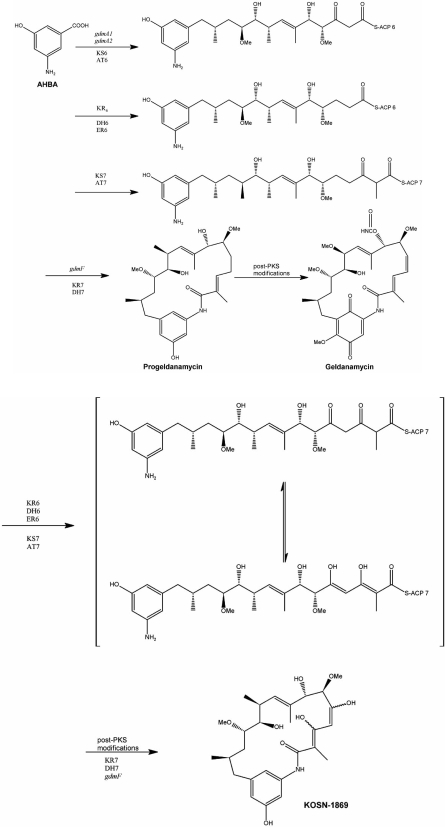
A. The series of biosynthetic reactions in *Streptomyces* leading to biosynthesis of the benzoquinone ansamycin geldanamycin. B. the ring closure reaction catalysed by an amide synthetase is highlighted. (After [58,59]). AHBA is amino hydroxybenzoic acid. The amide synthetase is encoded by the gene *gdmf.* The earlier genes in the cluster are numbered alphabetically. PKS is polyketide synthetase.

**Fig. (7). Alignment of putative Amide Synthetase protein sequences.**
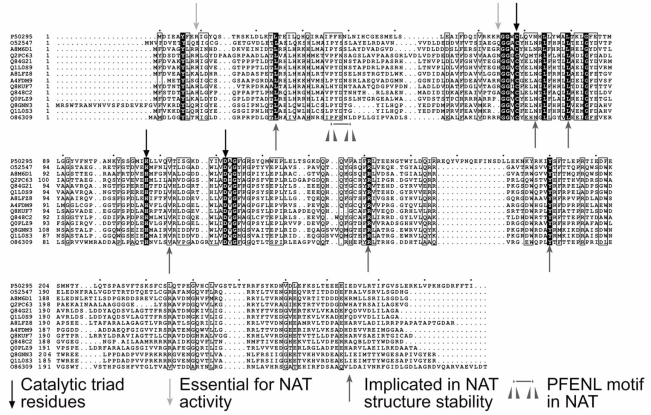
The sequences of the putative amide synthetases are shown in comparison with human NAT1 (P50295) and NAT from *Mycobacterium smegmatis* (086309). The essential residues as indicated are after [61]. The amide synthetases listed are as follows: O52547|RIFF_AMYMD 3-amino-5-hydroxybenzoic acid synthase - Amycolatopsis mediterranei (Nocardia mediterranei)., A8M6D1|A8M6D1_9ACTO N-acetyltransferase - Salinispora arenicola , Q2PC63|Q2PC63_STRAH Putative amide synthase - Streptomyces achromogenes subsp. Rubradiris, Q84G21|Q84G21_STRHY GdmF - Streptomyces hygroscopicus, Q1L0S9|Q1L0S9_STRHY GelD - Streptomyces hygroscopicus subsp. Duamyceticus, A8LFZ8|A8LFZ8_9ACTO N-acetyltransferase - Frankia sp. EAN1pec., A4FDM9|A4FDM9_SACEN 3-amino-5-hydroxybenzoic acid synthase - Saccharopolyspora erythraea (strain NRRL 23338), Q8KUF7|Q8KUF7_ACTPA Amide synthase - Actinosynnema pretiosum subsp. auranticum., Q848C2|Q848C2_STRHY Amide synthase - Streptomyces hygroscopicus (sequence cropped @ N-term to remove cloning artifacts)., Q0PLZ9|Q0PLZ9_9ACTO Putative N-acetyltransferase - Kitasatospora putterlickiae., Q8GNN3|Q8GNN3_STRHY ShnN - Streptomyces hygroscopicus., Q1L0S3|Q1L0S3_STRHY NapF - Streptomyces hygroscopicus subsp. duamyceticus.

**Table 1. Percent Conservation of Genes in Putative Nat-Containing Operon in M. tuberculosis H37Rv:** Rv3566a corresponds to a pseudogene, Rv3567c is thought to be hsaB and Rv3570c is thought to be *hsaA^1^*. N/A = not applicable for comparison as sequence is not present. Bioinformatic resources were as follows: [http://genolist.pasteur.fr/TubercuList/] and [http://www.tigr.org] and [http://www.sanger.ac.uk/Projects/M_bovis/]

Organisms Analysed	Percent Conservation with M. tuberculosis H37Rv (%)					
	*Rv3566c (nat)*	*Rv3566a*	*Rv3567c*	*Rv3568c(hsaC) *	*Rv3569c (hsaD)*	*Rv3570c*
*M. tuberculosis* CDC1551	100	100	100	100	100	100
*M. tuberculosis* Haarlem	100	100	100	100	100	100
*M. tuberculosis* F11	100	N/A	100	100	100	100
*M. tuberculosis* C	100	N/A	98	100	100	99
*M. bovis* BCG Pasteur	100	100	99.5	100	100	99.7
*M. bovis* AF2122/97	100	98.9	99	99.7	100	99.5
*M. marinum* M	75.4	N/A	90.5	90.7	89.9	90.9
*M. ulcerans* Agy99	75.8	N/A	89.9	90.7	89.5	90.6
*M. avium* K-10	67	N/A	88	89	89	90.6
*M. avium str.* 104	68	N/A	88	89	89	90.6
*M. *MCS	61	N/A	86	85	79	82
*M. *KMS	61	N/A	86	85	79	82
*M. *JLS	61	N/A	86	85	79	82
*M. smegmatis *mc^2^ 155	60	N/A	81	82	80	81
*M. vanbaalenii* PYR-1	59	N/A	82	82	77	81
*M. gilvum* PYR-GCK	60	N/A	82	82	77	81
*Rhodococcus* RHA1	43	N/A	74	81	75	78

1 After [26].

**Table 2. Comparison of Amino Acid Sequence Amongst NAT Homologues in the Mycolata:** The protein domains are based on the description in ^2^.

Organism	Percent Conservation with NAT from *M. tuberculosis* H37Rv (%)		
	Domain		
	1	2	3
*M .bovis* AF2122/97	100	100	100
*M. bovis* BCG Pasteur	100	100	100
*M. marinum*	82	75	67
*M. ulcerans*	82	75	67
*M. avium* str.104	68	69	64
*M. avium* K-10	68	68	64
*M.*MCS	69	57	56
*M.*KMS	69	57	56
*M.*JLS	68	58	57
*M. smegmatis *mc^2^ 155	62	61	56
*M. vanbaalenii* PYR-1	59	55	60
*M. gilvum *PYR-GCK	58	59	59
*Rhodococcus* RHA1	50	45	33

2 See [20].

## Footnotes

3 Sikora, A. L. and Blanchard, J. S. (2008). Kinetic mechanism and characterization of arylamine N-acetyltransferase from Mycobacterium tuberculosis. 235th ACS National Meeting, New Orleans, LA, USA.

4 Synthase and synthetase are used interchangeably.
